# CHIC Risk Stratification System for Predicting the Survival of Children With Hepatoblastoma: Data From Children With Hepatoblastoma in China

**DOI:** 10.3389/fonc.2020.552079

**Published:** 2020-11-18

**Authors:** Junting Huang, Yang Hu, Hong Jiang, Yanjie Xu, Suying Lu, Feifei Sun, Jia Zhu, Juan Wang, Xiaofei Sun, Juncheng Liu, Zijun Zhen, Yizhuo Zhang

**Affiliations:** ^1^State Key Laboratory of Oncology in South China, Collaborative Innovation Center for Cancer Medicine, Sun Yat-sen University Cancer Center, Guangzhou, China; ^2^Department of Pediatric Oncology, Sun Yat-sen University Cancer Center, Guangzhou, China; ^3^Department of Pediatric Surgery, First Affiliated Hospital of Sun Yat-sen University, Guangzhou, China

**Keywords:** hepatoblastoma, pediatric, risk stratification, prognostic factors, Childhood Hepatic Tumor International Consortium

## Abstract

**Objective:**

The aim of this study was to compare the accuracy of the Children’s Oncology Group (COG) risk stratification system to the Children’s Hepatic tumor International Collaboration (CHIC) risk stratification system for predicting the prognosis of Chinese children with hepatoblastoma (HB).

**Methods:**

Clinicopathological data of 86 patients diagnosed with HB between January 2014 and December 2017 were retrieved. The study endpoints were the 1- and 3-year overall survival (OS) and disease-free survival (DFS) were analyzed to evaluate the predictive value.

**Results:**

The 1-, 3-year OS and DFS of the 86 patients were 86.0%, 76.3%, and 74.4%, 54.0%, respectively. Univariate analyses revealed that age at diagnosis had a significant role in prognosis for both OS and DFS, along with PRETEXT staging and metastasis at diagnosis. Multivariate analysis showed that metastasis at diagnosis (HR 3.628, 95% CI 1.404-9.378, P=0.008), PRETEXT staging system (HR 2.176, 95% CI 1.230-3.849, P=0.008) and age at diagnosis (HR 2.268, 95% CI 1.033-4.982, P=0.041) were independent factors for OS. For DFS, the independent factors were the PRETEXT staging system (HR 2.241, 95% CI 1.533-3.277, P<0.001) and age at diagnosis (HR 1.792, 95% CI 1.018-3.154, P=0.043). Both COG and CHIC risk stratification systems could effectively predict the prognosis of children with HB for OS. For DFS, the CHIC risk stratification system was more effective. In addition, the CHIC risk stratification system had a higher c-index (OS 0.743, DFS 0.730), compared to the COG risk stratification system (OS 0.726, DFS 0.594).

**Conclusion:**

Age at diagnosis played a significant role in prognosis. Compared to the COG risk stratification system, the CHIC risk stratification system was superior in predicting the survival of Chinese children with HB.

## Introduction

HB is a rare childhood tumor with an annual incidence of 1.5 cases per million children, occurring predominantly in the first 2 years of life (1). Its overall survival in the past three decades has increased from ~30% to ~70% (2, 3); largely contributed due to improvement in imaging technology, surgical methods, timely surgeries, and chemotherapy prescription (4–6). However, milestone developments for the risk stratification of HB still lag behind more common pediatric cancers (7).

In the past decades, four separate cooperative multicenter trial groups have performed systematic treatment studies in HB, namely, the International Childhood Liver Tumor Strategy Group (SIOPEL); the Children’s Oncology Group (COG), and its legacy groups the Children’s Cancer Group (CCG) and the Pediatric Oncology Group (POG); the German Society for Pediatric Oncology and Haematology (GPOH); and the Japanese Study Group for Pediatric Liver Tumors (JPLT) (8). After accumulating and analyzing large amounts of data, these organizations proposed that the small cell undifferentiated (SCU) histology and low serum AFP at diagnosis could be associated with adverse outcomes in survival (9, 10) and have been constantly investigating novel prognostic factors (11–14). However, as the stratification criteria used in each group are different, it is not possible to compare the prognosis of each group clearly.

The small number of patients and the use of multiple different staging systems in the four main trial groups hindered the comparative evaluation of treatment outcomes in the pediatric HB trial. To solve this problem, CHIC has established a new international risk-stratified staging system by analyzing the database that includes comprehensive data from 1605 children treated in eight multicenter HB trials over 25 years (15). The new risk-stratified staging system—the Children’s Hepatic tumors International Collaboration—Hepatoblastoma Stratification (CHIC-HS) is shown as stratification trees based on four clinically familiar PRETEXT groups. CHIC-HS has not only confirmed the prognostic importance of factors such as metastatic disease, low AFP concentration (≤100 ng/ml), and PRETEXT group, that were originally proposed but also suggested additional new prognostic factors and more detailed grouping criteria (7).

This study aimed to compare the prognostic ability of the COG risk stratification system (**Table S1**) and the CHIC risk stratification system for prognosticating the survival of Chinese children with HB (**Figure S1**).

## Patients and Methods

### Ethics Statement

The research was approved by the institutional review board (IRB) of Sun Yat-sen University Cancer Center (SYSUCC; Guangzhou, China), the IRB number is B2019-232-01, and written informed consent was obtained from each patients’ parents involved in the study. All the original data was deposited on http://www.researchdata.org.cn (RDD number RDDA2020001362).

### Patients

Patients who were diagnosed as HB from January 2014 to December 2017 at the SYSUCC and the First Affiliated Hospital of Sun Yat-sen University (Guangzhou, China) were identified from our prospective database.

Patients who met all of the following criteria were included in the study analyses: 1) newly diagnosed patients; 2) age less than 18 years old; 3) histologic confirmation of HB. Since the current study aimed to analyze the prognostic factors for long-term survival, we excluded patients who died <30 days following surgery or died of chemotherapy side effects.

At the time of initial diagnosis, all the parameters mentioned by risk-stratified staging system were recorded and evaluated as possible predictors of survivals including age, alpha-fetoprotein (AFP), metastatic disease (M), macrovascular involvement of all hepatic veins (V), or portal bifurcation (P), contiguous extrahepatic tumor (E), multifocal tumor (F), and spontaneous rupture (R), tumor size and pathological pattern. Tumor size and VPEFRM were judged by contrast abdominal computed tomography (CT), abdominal ultrasound scan and intraoperative conditions. In general, P1 and P2 were defined as P+, V1, V2, and V3 were defined as V+ (16). None of the patients underwent liver transplantation.

### Chemotherapy

According to the COG risk stratification system, the chemotherapy regimens of each group were different. Chemotherapy was not performed in the very low-risk group. The C5V(cisplatin, 5-FU, vincristine) chemotherapy regimen was used in the low-risk group for a total of four to six courses. Forty-five patients were defined as intermediate risk, of whom 30 patients were treated with the C5V(cisplatin, 5-FU, vincristine)/PLADO(cisplatin, doxorubicin) chemotherapy regimen, eight patients were treated with the PLADO(cisplatin, doxorubicin) chemotherapy regimen, and seven patients were treated with the C5VD (cisplatin, 5-FU, vincristine, doxorubicin) chemotherapy regimen for a total of six to eight courses. The C5VD (cisplatin, 5-FU, vincristine, doxorubicin)/IIV (ifosfamide, irinotecan, vincristine) chemotherapy regimen was used in the high-risk group for a total of eight courses (**Tables S2, S3**). Alpha-fetoprotein (AFP) was assessed before each course of chemotherapy and tumor efficacy (17) was evaluated every two courses (CT or MR assessment).

### Follow-Up

After the end of treatment, patients were followed-up with a comprehensive review included alpha-fetoprotein (AFP), complete blood routine, biochemical routine, electrocardiogram, echocardiography, chest and CT abdomen and so on. In the first year, patients were followed every 3 months based on assessment of their alpha-fetoprotein (AFP) level, complete blood and biochemical (liver and kidney function and electrolytes) routine tests, abdominal ultrasound, chest X-ray, and electrocardiogram. Abdominal CT and echocardiogram were reviewed every 6 months. Patients were re-examined every 6 months from the second year and every year from the third year. When a metastasis was suspected, a chest CT scan, bone scintigraphy, positron emission tomography (PET), and biopsy (if necessary) were performed to confirm the metastasis and/or recurrence. The last follow-up date for patients who were still alive was August 2019.

Causes of death and sites of recurrence were determined from death certificates, medical interviews, and radiological findings. The study endpoints were overall survival (OS), defined as the time period from the date of initial diagnosis until death or last follow-up, and disease-free survival (DFS), defined as the time period from the date of initial diagnosis until occurrence of an event (recurrence, progressive disease, death, or diagnosis of a second malignant neoplasm) or last contact, whichever occurred first.

### Statistical Analyses

The statistical analyses except C-index analyses were performed using the SPSS software, version 19.0 (SPSS Company, Chicago, Illinois, USA). The OS and DFS were calculated by the Kaplan–Meier method and compared using the log-rank test. The prognostic varieties in predicting OS and DFS were assessed by multivariate Cox proportional hazards regression analyses. All covariates that had clinical significance were included in a multivariate Cox proportional hazards model. Results were given as mean ± S.D. All statistical tests were two-sided, and a significant difference was considered for P < 0.05. The concordance index method (c-index) was used to rank the different risk-stratified staging systems according to their capacity of discriminating patients according to the outcome. The c-index was calculated using R with the rms package (18) of Dr. Frank Harrell.

## Results

### Baseline Characteristics

A total of 100 patients who were diagnosed as HB were identified during that period. Based on our inclusion criteria, 14 patients were excluded from this study, of whom two died during the perioperative period (2%, 2/100), three died while on chemotherapy (3%,3/100), and nine who were lost in follow-up (9%, 9/100). The patients’ baseline characteristics are summarized in **Table 1**. A total of 86 patients were included in the study, comprising of 54 (62.8%) male and 32 (37.2%) female, with a median age at diagnosis of 26.94 months (range: 0.9–139.6 months). AFP was elevated (>100 ng/ml or age-specific upper limit) in 84 (97.7%) patients. Based on the results of the imaging studies, patients were classified using the PRETEXT staging system and judged whether they had distant metastases and VPEFR. 22 (25.6%) patients had distant metastasis and 44 (51.2%) were positive for VPEFR at diagnosis. Fetal histology (n=29) was the most common pathological subtype in our patients on postoperative pathological results. All patients were classified using the COG risk group and CHIC risk group. Using the COG risk stratification system, 3 (3.5%) patients were considered as having very low-risk, 5 (5.8%) as low-risk, 45 (52.3%) as intermediate-risk, and 33 (38.4%) as high-risk. Using the CHIC risk stratification system, five (5.8%) patients were considered as having very low risk, 13 (15.1%) as low risk, 38 (44.2%) as intermediate risk, and 33 (38.4) as high risk.

**Table 1:** Characteristics of patients included in the study.

		N	%
ALL		86	100
Gender	Male	54	62.8
	Female	32	37.2
Age(months)	<36	51	59.3
	36–96	31	36.0
	≥96	4	4.7
AFP at diagnosis (ng/ml)	≤100	2	2.3
	101–1000	7	8.1
	>1000	77	89.5
PRETEXT staging	I	9	10.5
	II	38	44.2
	III	14	16.3
	IV	25	29.1
Metastasis at diagnosis	No	64	74.4
	Yes	22	25.6
VPEFR	VPEFR (+)	44	51.2
	VPEFR (-)	42	48.8
	V (+)	9	10.5
	P (+)	7	8.1
	E (+)	23	26.7
	F (+)	15	17.4
	R (+)	5	5.8
Histological subtype	Epithelial	4	4.7
	Fetal(Pure Fetal excluded)	29	33.7
	Pure Fetal	2	2.3
	Embryonal	5	5.8
	Mixed embryonal–fetal	7	8.1
	Mixed epithelial–mesenchymal	22	25.6
	HB (unknown)	17	19.8
COG risk group	Very low risk	3	3.5
	Low risk	5	5.8
	Intermediate risk	45	52.3
	High risk	33	38.4
CHIC risk group	Very low risk	5	5.8
	Low risk	13	15.1
	Intermediate risk	38	44.2
	High risk	30	34.9

### Univariate Analysis of Prognostic Factors

All factors listed in **Table 2** were included in univariate analysis (**Table 2**), of which, age at diagnosis (OS P=0.029, DFS P=0.027), PRETEXT staging (OS P<0.001, DFS P<0.001), and metastasis at diagnosis (OS P<0.001, DFS P=0.007) were identified as significant prognostic factors for OS and DFS. For OS, the 3-year OS of patients younger than 36 months, aged from 36 months to 96 months and older than 96 months were 83.4%, 67.6%, and 50.0%, respectively. The 3-year OS of the PRETEXT stage I, II, III, and IV were 87.5%, 91.4%, 71.4%, and 52.0%. The 3-year OS of M (+) and M (-) were 44.4% and 87.4%. For DFS, the 3-year DFS of patients from different age groups were 64.7%, 35.9%, and 50.0%, respectively. The 3-year DFS of the PRETEXT stage I, II, III, and IV were 87.5%, 71.6%, 42.9%, and 20.0%. The 3-year DFS of M (+) and M (-) were 29.2% and 63.0%.

**Table 2:** Univariate analysis of prognostic factors.

	N	Overall survival		Disease-free survival	
		3-year OS (%)	P	3-year DFS (%)	P
Gender					
Male	54	79.0	0.551	54.7	0.663
Female	32	71.7		53.1	
Age at diagnosis(months)					
<36	51	83.4	0.029	64.7	0.027
36–96	31	67.6		35.9	
≥96	4	50.0		50.0	
AFP at diagnosis (ng/ml)					
≤100	2	100	0.26	100	0.563
101–1000	7	100		35.7	
>1000	77	73.7		53.3	
PRETEXT staging					
I	9	87.5	<0.001	87.5	<0.001
II	38	91.4		71.6	
III	14	71.4		42.9	
IV	25	52.0		20.0	
Metastasis at diagnosis					
Yes	22	44.4	<0.001	29.2	0.007
No	64	87.4		63.0	
VPEFR					
VPEFR (+)	44	72.7	0.401	50.6	0.327
VPEFR (-)	42	79.8		57.0	
Histological subtype					
Pure Fetal	2	50.0	0.497	50.0	0.900
Other subtype	84	77.0		54.1	

### Multivariate Analysis of Prognostic Factors

Multivariate analysis showed that metastasis at diagnosis (HR 3.628, 95% CI 1.404-9.378, P=0.008), PRETEXT staging (HR 2.176, 95% CI 1.230-3.849, P=0.008) and age at diagnosis (HR 2.268, 95% CI 1.033-4.982, P=0.041) were independent prognostic factors for OS (**Table 3**). However, AFP at diagnosis, VPEFR and histological subtype were not (P>0.05) for OS. For DFS, multivariate analysis showed that PRETEXT staging (HR 2.241, 95% CI 1.533-3.277, P<0.001) and age at diagnosis (HR 1.792, 95% CI 1.018-3.154, P=0.043) were independent prognostic factors. However, metastasis at diagnosis, AFP at diagnosis, VPEFR and histological subtype were not (P>0.05) for DFS (**Table 3**).

**Table 3:** Multivariate analysis of prognostic factors.

Variables	HR	95.0% CI	P value
Overall survival			
Metastasis at diagnosis	3.628	1.404–9.378	0.008
PRETEXT staging	2.176	1.230–3.849	0.008
Age at diagnosis	2.268	1.033–4.982	0.041
Disease-free survival			
PRETEXT staging	2.241	1.533–3.277	<0.001
Age at diagnosis	1.792	1.018–3.154	0.043

### Survival

**Figure 1:**
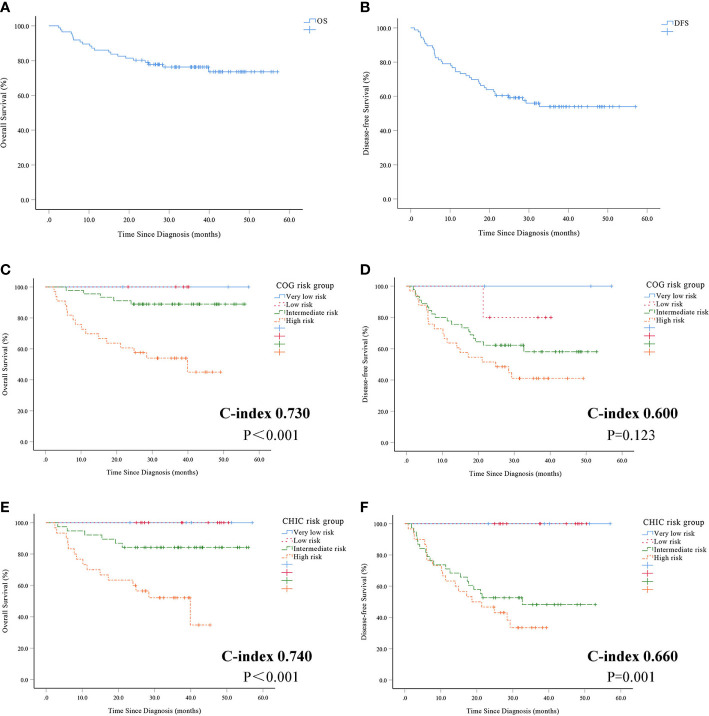
Overall survival **(A)** and disease-free survival **(B)** in 86 patients with hepatoblastoma. Overall survival **(C)** and disease-free survival **(D)** using COG risk stratification system. Overall survival **(E)** and disease-free survival **(F)** using CHIC risk stratification system.

The median follow-up period was 33.9 months (range 2.4–57.0 months). The 1-, 3-year OS of the 86 investigated patients were 86.0%, 76.3%, respectively (**Figure 1A**). The median OS was 33.9 months. The 1-, 3-year DFS were 74.4%, 54.0%, respectively (**Figure 1B**). The median DFS was 26.6 months.

There was a statistically significant difference between patients with very low-risk, low-risk, intermediate-risk and high-risk based on the COG risk stratification system for OS (P<0.001, **Figure 1C**). But statistically significant differences were not observed between patients using the COG risk stratification system for DFS (P = 0.123, **Figure 1D**). There was a statistically significant difference between patients with very low-risk, low-risk, intermediate-risk and high-risk using the CHIC risk stratification system for both OS (P<0.001, **Figure 1E**) and DFS (P = 0.001, **Figure 1F**).

The prognostic value of the COG and CHIC risk stratification system was compared by means of Harrell’s concordance index. The CHIC risk stratification system had a higher c-index (OS 0.740, DFS 0.660) compared to the COG risk stratification system (OS 0.730, DFS 0.600).

## Discussion

Based on the proposal of CHIC risk stratification system (7), we compared the accuracy of the COG risk stratification system to the CHIC risk stratification system for predicting the prognosis of Chinese children with HB. First of all, our results showed that the 1-, 3-year OS of children with HB were 86.0%, 76.3%, and 74.4%, 54.0% in DFS, respectively. What’s more, our study indicated that age at diagnosis played a significant role in prognosis for both OS and DFS, along with the PRETEXT staging and metastasis at diagnosis. Above all, our results revealed that compared to the COG risk stratification system we used, the CHIC risk stratification system was better to predict the survival in children with HB, especially for DFS.

With the use of adjuvant and neo-adjuvant chemotherapy, the 1-, 3-year OS and DFS were 86.0%, 76.3%, and 74.4%, 54.0% in our cohort, respectively. The literature on the treatment outcome of children with HB is inadequate. Chinese Children’s Cancer Group (19) (CCCG) reported 6-year OS of standard risk and high risk groups were 90.3% ± 2.9% and 67.6 ± 7.0%. Liu et al. (20) revealed 5-year OS and DFS of children with HB in Hong Kong were 77.6% ± 5.5% and 69.2% ± 6.1%, respectively. Qiao et al. (21) showed the 5-year OS and EFS rates were 63.4% and 57.1%. Recent clinical trials initiated by four major organizations generally reported that the 3-year OS was above 80% (22). The SIOPEL-4 (23) results suggested that 3-year OS of children with high-risk HB was 83% and EFS was 76%. The JPLT-2 (24, 25) study of 279 patients indicated the 3-, 5-year OS were 82.4%, 80.9%, and 69.9%, 62.4% in DFS, respectively. Results of the GPOH (26) Study HB99 showed the 5-year OS was 58% for high risk patients and 94% for standard risk patients.

Similarly, our study also showed unsatisfactory results that the 3-year OS and DFS were 54.0%, 41.0% for the COG high-risk group, and 52.1%, 33.5% for the CHIC high-risk group. The survival of patients in high risk group was gloomy, especially in DFS. First of all, due to economic and medical limitations, all high-risk patients had not undergone liver transplantation. Some data suggested that the overall long-term outcome of primary liver transplantation for HB was excellent (27). Secondly, the chemotherapy regimen-C5VD/IIV for high-risk patients in this study was derived from a clinical trial, which was terminated in advance due to poor efficacy. In other words, our treatment of high-risk patients had failed. In addition, the lack of molecular variables at first diagnosis affected our judgment of prognosis. Therefore, it’s necessary to seek more effective treatment to improve the survival of patients in high-risk groups.

Compared to the COG risk stratification system, the CHIC risk stratification system excluded pathologic subtypes and included age at diagnosis to form stratification trees based on four clinically familiar PRETEXT groups. Over the past 10 years, four major organizations have been devoted to exploring effective prognostic factors in children with HB (23, 24, 26–29). In 2013, based on a database involving 1605 HB cases treated between 1988 and 2008 from eight completed HB trials, CHIC identified novel prognostic factors for HB (15). Advanced PRETEXT group, macrovascular venous or portal involvement, contiguous extrahepatic disease, primary tumor multifocality, and tumor rupture significantly increased risk for EFS-event. Higher age (≥8 years), low AFP (<100 ng/ml) and metastatic disease significantly increased risk for OS-event. Recently, CHIC stressed that risk of an event for children with HB increased with increasing age at diagnosis (30). In China, several results about the prognostic factors of children with HB have appeared recently. Qiao et al. (21, 31) presented AFP <100 or >1000 (ng/ml), multifocality, vascular invasion, metastases, and PRETEXT stage were associated with poor prognosis. Another multicentric study demonstrated that female, age over 5 years, PRETEXT III, PRETEXT IV, incomplete surgical resection, and with metastasis had poorer prognosis (19). Our findings confirmed that age at diagnosis played a significant role in prognosis for both OS and DFS, along with the PRETEXT staging and metastasis at diagnosis. Although AFP at diagnosis, VPEFR, and histological subtype did not show statistical differences in univariate analysis and multivariate analysis, but we cannot deny that these were acknowledged factors associated with prognosis. In our cohort, only 2 (2.3%) patients had low AFP (≤100 ng/ml) at diagnosis and no one was confirmed to be SCU histologic subtype. The absence of the above cases may be the reason for the non-statistical difference in AFP at diagnosis and pathological type.

Our results showed that both COG and CHIC risk stratification systems could effectively predict the prognosis of children with HB for OS. For DFS, the CHIC risk stratification system was effective, but the COG risk stratification system was not. In addition, the CHIC risk stratification system had a higher c-index (OS 0.743, DFS 0.730), compared to the COG risk stratification system (OS 0.726, DFS 0.594). Yoon et al. (32) provided supporting evidence of utility of the CHIC risk stratification system by providing good prognostic value of its variables. The study from Hong Kong also demonstrated the CHIC risk stratification system was effective to classify different risk groups for predicting the prognosis (20). However, the results clearly indicated that both the COG and CHIC risk stratification system did not had good efficacy to distinguish between the prognosis of very low-risk group and low-risk group. Therefore, the risk stratification system more suitable for children with HB in China needs further development.

Our results confirmed good prognostic value of age at diagnosis, the PRETEXT staging and metastasis at diagnosis. More importantly, our study validated the feasibility of the risk stratification system proposed by CHIC and its application to Chinese children with HB. Although retrospective, the data we have presented were based on a relatively long duration of follow-ups (median, 33.9 months) from the children who were diagnosed as HB from January 2014 to December 2017.We also used sensitive and specific imaging technologies and histological confirmation in order for us to provide a valid base to evaluate the prognostic value of the risk stratifications. However, there were some limitations in the present study that should be commented. Firstly, it is a single-institution study of a fairly homogenous population. Due to the rarity of HB, the inadequate number of cases may be difficult to define several prognostic factors, such as AFP at diagnosis. Secondly, patients with intermediate-risk, were offered different chemotherapy regimens which might have contributed to the differences in patients’ prognosis. Thirdly, our study did not analyze the effect of molecular variables which would be potential prognostic factors alternative to clinical factors in the future. Several prognostic markers have been reported in associated with HB (22, 33). Sumazin et al. demonstrated high NFE2L2 activity and high expression of LIN28B, HMGA2, SALL4 were associated with poor prognosis and marked high‐risk group (34). Another research indicated that the four novel tumor suppressor candidates including GPR180, MST1R, OCIAD2, and PARP6 were potentially useful molecular markers for predicting poor prognosis in HB patients (35). In the future studies, we should try to include genetic mutation in the prognostic correlation analysis.

## Conclusion

In this cohort of Chinese children with HB, age at diagnosis showed a significant association with patient outcome, along with the PRETEXT staging and metastasis at diagnosis. Compared to the COG risk stratification system, the CHIC risk stratification system was superior in predicting the survival of Chinese children with HB.

## Data Availability Statement

The datasets presented in this study can be found in online repositories. The names of the repository/repositories and accession number(s) can be found below: http://www.researchdata.org.cn (RDD number RDDA2020001362).

## Ethics Statement

The studies involving human participants were reviewed and approved by the institutional review board (IRB) of Sun Yat-sen University Cancer Center (SYSUCC; Guangzhou, China). Written informed consent to participate in this study was provided by the participants’ legal guardian/next of kin. Written informed consent was obtained from the minor(s)’ legal guardian/next of kin for the publication of any potentially identifiable images or data included in this article.

## Author Contributions

JH, YH, XS, YZ, JL, and ZZ contributed conception and design of the study. HJ, YX, and SL organized the database. FS, JZ, and JW performed the statistical analysis. JH and YH wrote the first draft of the manuscript. HJ, YZ, JL, and ZZ wrote sections of the manuscript. All authors contributed to the article and approved the submitted version.

## Conflict of Interest

The authors declare that the research was conducted in the absence of any commercial or financial relationships that could be construed as a potential conflict of interest.

## References

[1] Spector LG Birch J The epidemiology of hepatoblastoma. Pediatr Blood Cancer (2012) 59:776–9. 10.1002/pbc.24215 22692949

[2] Sharma D Subbarao G Saxena R Hepatoblastoma. Semin Diagn Pathol (2017) 34:192–200. 10.1053/j.semdp.2016.12.015 28126357

[3] Feng J Polychronidis G Heger U Frongia G Mehrabi A Hoffmann K Incidence trends and survival prediction of hepatoblastoma in children: a population-based study. Cancer Commun (Lond) (2019) 39:62. 10.1186/s40880-019-0411-7 31651371 PMC6813130

[4] Perilongo G Maibach R Shafford E Brugieres L Brock P Morland B Cisplatin versus cisplatin plus doxorubicin for standard-risk hepatoblastoma. N Engl J Med (2009) 361:1662–70. 10.1056/NEJMoa0810613 19846851

[5] Zsiros J Brugieres L Brock P Roebuck D Maibach R Child M Efficacy of irinotecan single drug treatment in children with refractory or recurrent hepatoblastoma–a phase II trial of the childhood liver tumour strategy group (SIOPEL). Eur J Cancer (2012) 48:3456–64. 10.1016/j.ejca.2012.06.023 22835780

[6] Hishiki T Watanabe K Ida K Hoshino K Iehara T Aoki Y The role of pulmonary metastasectomy for hepatoblastoma in children with metastasis at diagnosis: Results from the JPLT-2 study. J Pediatr Surg (2017) 52:2051–5. 10.1016/j.jpedsurg.2017.08.031 28927977

[7] Meyers RL Maibach R Hiyama E Haberle B Krailo M Rangaswami A Risk-stratified staging in paediatric hepatoblastoma: a unified analysis from the Children’s Hepatic tumors International Collaboration. Lancet Oncol (2017) 18:122–31. 10.1016/S1470-2045(16)30598-8 PMC5650231 27884679

[8] Perilongo G Malogolowkin M Feusner J Hepatoblastoma clinical research: lessons learned and future challenges. Pediatr Blood Cancer (2012) 59:818–21. 10.1002/pbc.24217 22678761

[9] De Ioris M Brugieres L Zimmermann A Keeling J Brock P Maibach R Hepatoblastoma with a low serum alpha-fetoprotein level at diagnosis: the SIOPEL group experience. Eur J Cancer (2008) 44:545–50. 10.1016/j.ejca.2007.11.022 18166449

[10] Trobaugh-Lotrario AD Tomlinson GE Finegold MJ Gore L Feusner JH Small cell undifferentiated variant of hepatoblastoma: adverse clinical and molecular features similar to rhabdoid tumors. Pediatr Blood Cancer (2009) 52:328–34. 10.1002/pbc.21834 PMC2946187 18985717

[11] Meyers RL Rowland JR Krailo M Chen Z Katzenstein HM Malogolowkin MH Predictive power of pretreatment prognostic factors in children with hepatoblastoma: a report from the Children’s Oncology Group. Pediatr Blood Cancer (2009) 53:1016–22. 10.1002/pbc.22088 PMC4408767 19588519

[12] Aronson DC Schnater JM Staalman CR Weverling GJ Plaschkes J Perilongo G Predictive value of the pretreatment extent of disease system in hepatoblastoma: results from the International Society of Pediatric Oncology Liver Tumor Study Group SIOPEL-1 study. J Clin Oncol (2005) 23:1245–52. 10.1200/JCO.2005.07.145 15718322

[13] Fuchs J Rydzynski J Von Schweinitz D Bode U Hecker H Weinel P Pretreatment prognostic factors and treatment results in children with hepatoblastoma: a report from the German Cooperative Pediatric Liver Tumor Study HB 94. Cancer-Am Cancer Soc (2002) 95:172–82. 10.1002/cncr.10632 12115331

[14] Maibach R Roebuck D Brugieres L Capra M Brock P Dall’Igna P Prognostic stratification for children with hepatoblastoma: the SIOPEL experience. Eur J CANCER (2012) 48:1543–9. 10.1016/j.ejca.2011.12.011 22244829

[15] Czauderna P Haeberle B Hiyama E Rangaswami A Krailo M Maibach R The Children’s Hepatic tumors International Collaboration (CHIC): Novel global rare tumor database yields new prognostic factors in hepatoblastoma and becomes a research model. Eur J Cancer (2016) 52:92–101. 10.1016/j.ejca.2015.09.023 26655560 PMC5141607

[16] Roebuck DJ Aronson D Clapuyt P Czauderna P de Ville DGJ Gauthier F 2005 PRETEXT: a revised staging system for primary malignant liver tumours of childhood developed by the SIOPEL group. Pediatr Radiol (2007) 37:123–32; quiz 249-50. 10.1007/s00247-006-0361-5 17186233 PMC1805044

[17] Zsiros J Maibach R Shafford E Brugieres L Brock P Czauderna P Successful treatment of childhood high-risk hepatoblastoma with dose-intensive multiagent chemotherapy and surgery: final results of the SIOPEL-3HR study. J Clin Oncol (2010) 28:2584–90. 10.1200/JCO.2009.22.4857 20406943

[18] Huang J Zhang Y Peng Z Gao H Xu L Jiao LR A modified TNM-7 staging system to better predict the survival in patients with hepatocellular carcinoma after hepatectomy. J Cancer Res Clin Oncol (2013) 139:1709–19. 10.1007/s00432-013-1497-y PMC11824667 23982274

[19] Yuan XJ Wang HM Jiang H Tang MJ Li ZL Zou X Multidisciplinary effort in treating children with hepatoblastoma in China. Cancer Lett (2016) 375:39–46. 10.1016/j.canlet.2016.02.051 26945966

[20] Liu A Ip J Leung A Luk CW Li CH Ho K Treatment outcome and pattern of failure in hepatoblastoma treated with a consensus protocol in Hong Kong. Pediatr Blood Cancer (2019) 66:e27482. 10.1002/pbc.27482 30270490

[21] Qiao GL Li L Cheng W Ge J Zhang Z Wei Y Predictors of survival after resection of children with hepatoblastoma: A single Asian center experience. Eur J Surg Oncol (2014) 40:1533–9. 10.1016/j.ejso.2014.07.033 25103357

[22] Czauderna P Lopez-Terrada D Hiyama E Haberle B Malogolowkin MH Meyers RL Hepatoblastoma state of the art: pathology, genetics, risk stratification, and chemotherapy. Curr Opin Pediatr (2014) 26:19–28. 10.1097/MOP.0000000000000046 24322718

[23] Zsiros J Brugieres L Brock P Roebuck D Maibach R Zimmermann A Dose-dense cisplatin-based chemotherapy and surgery for children with high-risk hepatoblastoma (SIOPEL-4): a prospective, single-arm, feasibility study. Lancet Oncol (2013) 14:834–42. 10.1016/S1470-2045(13)70272-9 PMC3730732 23831416

[24] Hiyama E Hishiki T Watanabe K Ida K Ueda Y Kurihara S Outcome and Late Complications of Hepatoblastomas Treated Using the Japanese Study Group for Pediatric Liver Tumor 2 Protocol. J Clin Oncol (2020) 38:2488–98. 10.1200/JCO.19.01067 32421442

[25] Hishiki T Matsunaga T Sasaki F Yano M Ida K Horie H Outcome of hepatoblastomas treated using the Japanese Study Group for Pediatric Liver Tumor (JPLT) protocol-2: report from the JPLT. Pediatr Surg Int (2011) 27:1–8. 10.1007/s00383-010-2708-0 20922397

[26] Haberle B Maxwell R Schweinitz DV Schmid I High Dose Chemotherapy with Autologous Stem Cell Transplantation in Hepatoblastoma does not Improve Outcome. Results of the GPOH Study HB99. Klin Padiatr (2019) 231:283–90. 10.1055/a-1014-3250 31645068

[27] Brown J Perilongo G Shafford E Keeling J Pritchard J Brock P Pretreatment prognostic factors for children with hepatoblastoma– results from the International Society of Paediatric Oncology (SIOP) study SIOPEL 1. Eur J Cancer (2000) 36:1418–25. 10.1016/s0959-8049(00)00074-5 10899656

[28] Malogolowkin MH Katzenstein HM Meyers RL Krailo MD Rowland JM Haas J Complete surgical resection is curative for children with hepatoblastoma with pure fetal histology: a report from the Children’s Oncology Group. J Clin Oncol (2011) 29:3301–6. 10.1200/JCO.2010.29.3837 PMC3158601 21768450

[29] Haeberle B Schweinitz D Treatment of hepatoblastoma in the German cooperative pediatric liver tumor studies. Front Biosci (Elite Ed) (2012) 4:493–8. 10.2741/395 22201890

[30] Haeberle B Rangaswami A Krailo M Czauderna P Hiyama E Maibach R The importance of age as prognostic factor for the outcome of patients with hepatoblastoma: Analysis from the Children’s Hepatic tumors International Collaboration (CHIC) database. Pediatr Blood Cancer (2020) 67:e28350. 10.1002/pbc.28350 32383794

[31] Qiao GL Chen Z Wang C Ge J Zhang Z Li L Pure fetal histology subtype was associated with better prognosis of children with hepatoblastoma: A Chinese population-based study. J Gastroenterol Hepatol (2016) 31:621–7. 10.1111/jgh.13165 26401976

[32] Yoon HM Hwang J Kim KW Namgoong JM Kim DY Koh KN Prognostic Factors for Event-Free Survival in Pediatric Patients with Hepatoblastoma Based on the 2017 PRETEXT and CHIC-HS Systems. Cancers (Basel) (2019) 11(9):1387. 10.3390/cancers11091387 PMC6769992 31540387

[33] Meyers RL Tiao G de Ville DGJ Superina R Aronson DC Hepatoblastoma state of the art: pre-treatment extent of disease, surgical resection guidelines and the role of liver transplantation. Curr Opin Pediatr (2014) 26:29–36. 10.1097/MOP.0000000000000042 24362406

[34] Sumazin P Chen Y Trevino LR Sarabia SF Hampton OA Patel K Genomic analysis of hepatoblastoma identifies distinct molecular and prognostic subgroups. Hepatology (2017) 65:104–21. 10.1002/hep.28888 27775819

[35] Honda S Minato M Suzuki H Fujiyoshi M Miyagi H Haruta M Clinical prognostic value of DNA methylation in hepatoblastoma: Four novel tumor suppressor candidates. Cancer Sci (2016) 107:812–9. 10.1111/cas.12928 PMC4968605 26991471

