# Supplementary figures and images for: CHIC Risk Stratification System for Predicting the Survival of Children With Hepatoblastoma: Data From Children With Hepatoblastoma in China
Source: Front Oncol. 2020 Nov 18;10:552079. doi: 10.3389/fonc.2020.552079 (PMC7708347; doi:10.3389/fonc.2020.552079)

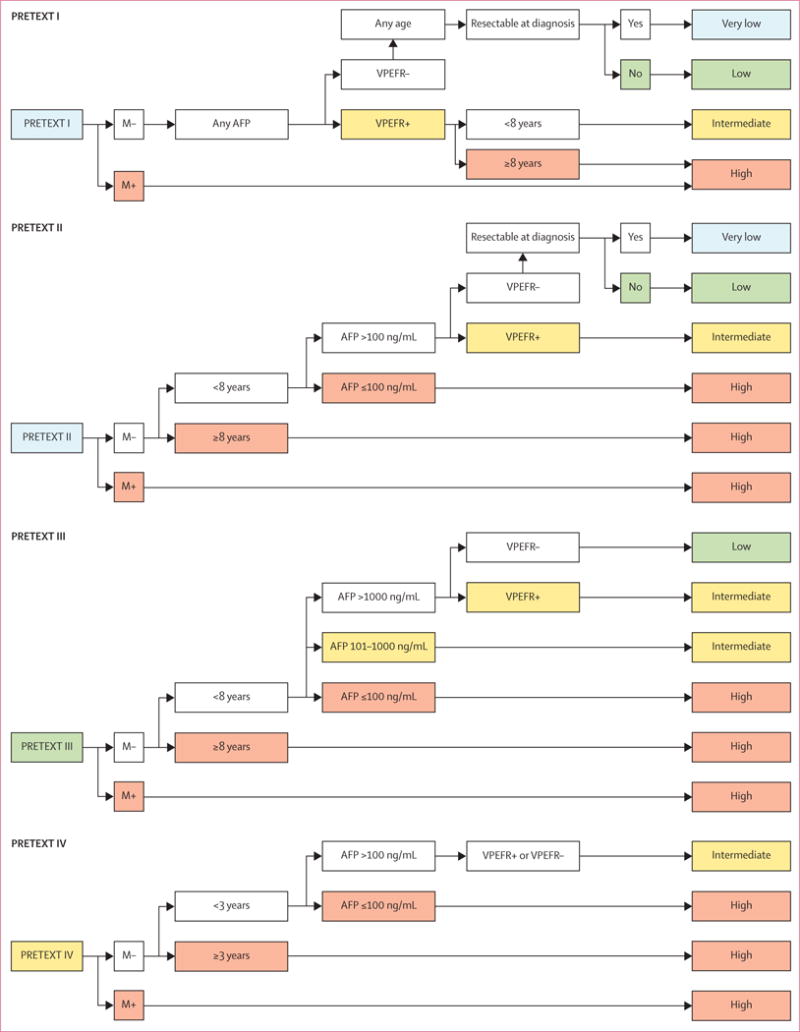

Supplement: Supplementary file 1 [file Image_1.jpeg]
