# Supplementary material for: CHIC Risk Stratification System for Predicting the Survival of Children With Hepatoblastoma: Data From Children With Hepatoblastoma in China
Source: Front Oncol. 2020 Nov 18;10:552079. doi: 10.3389/fonc.2020.552079 (PMC7708347; doi:10.3389/fonc.2020.552079)
Supplement: Supplementary file 2 [file Table_1.docx]

**Table S1** Risk stratification system for the Children's Oncology Group (COG)

| Risk group | Standard |
| --- | --- |
| **Very low risk** | PRETEXT I/II, pure fetal histology, and primary resection |
| **Low risk** | PRETEXT I/II, any histology, primary resection |
| **Intermediate risk** | PRETEXT II/III,IV, unresectable at diagnosis V+, P+, E+ |
| **High risk** | Any PRETEXT, M+, AFP<100ng/ml |
